# Impact of chronic diseases on effect of breast cancer screening

**DOI:** 10.1002/cam4.3036

**Published:** 2020-04-06

**Authors:** Anna‐Belle Beau, George M. Napolitano, Marianne Ewertz, Ilse Vejborg, Walter Schwartz, Per K. Andersen, Elsebeth Lynge

**Affiliations:** ^1^ Section of Environmental Health Department of Public Health University of Copenhagen Copenhagen Denmark; ^2^ Department of Oncology Odense University Hospital Institute of Clinical Research University of Southern Denmark Odense Denmark; ^3^ Department of Radiology Copenhagen University Hospital (Rigshospitalet) Copenhagen Denmark; ^4^ Mammography Centre Odense University Hospital Odense Denmark; ^5^ Section of Biostatistics Department of Public Health University of Copenhagen Copenhagen Denmark; ^6^ Centre for Epidemiological Research Nykøbing Falster Hospital Nykøbing Falster Denmark

**Keywords:** breast cancer mortality reduction, Breast cancer screening, chronic diseases, impact of screening, personalized screening, register‐based study

## Abstract

**Background:**

Although breast cancer screening reduces breast cancer mortality at the population level, subgroups of women may benefit differently. We investigated the impact of health status on the effect of breast cancer screening.

**Methods:**

The study included 181 299 women invited in two population‐based screening programs in Denmark and 1 526 446 control subjects, followed from April 1981 to December 2014. Poisson regressions were used to compare the observed breast cancer mortality rate in women invited to screening with the expected rate in the absence of screening among women with and without chronic diseases. Chronic diseases were defined as any diagnosis in the Charlson Comorbidity Index during 4 years before the first invitation to screening.

**Results:**

Almost 10% of women had chronic diseases before first invitation to screening. Whereas we observed a reduction in breast cancer mortality following invitation to screening of 28% (95% CI, 20% to 35%) among women without chronic diseases, only a 7% (95% CI, −39% to 37%) reduction was seen for women with chronic diseases (*P*‐value for interaction = .22). For participants, the reduction, corrected for selection bias, was 35% (95% CI 16% to 49%) for women without, and 4% (95% CI −146% to 62%) for women with chronic diseases (*P*‐value for interaction = .43).

**Conclusion:**

Our data indicate a marginal effect of mammography screening on breast cancer mortality in women with chronic diseases. If our results are confirmed in other populations, the presence of chronic diseases will be an important factor to take into consideration in personalized screening.

## Graphical abstract

Although breast cancer screening reduces breast cancer mortality at the population level, subgroups of women may benefit differently. The paper estimated the effect of screening on breast cancer mortality for women divided by the presence or absence of chronic diseases at the time of invitation to screening. Our results indicate that breast cancer screening might have only a marginal impact on breast cancer mortality in women with chronic diseases.

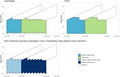

## 1 INTRODUCTION

Early detection of breast cancer by mammography screening has reduced breast cancer mortality. In both the United States and Europe, official guidelines recommend biennial screening for women aged 50 to 69 or 74 years, but starting age at 40 years is also quite common.^1^, ^2^ At the population level, we show a 20% reduction in breast cancer mortality in Danish women invited to a population‐based screening program.^3^ Nevertheless, subgroups of women may contribute very differently to this average.

Attempts are currently made to move screening from the “one model fits all” to more personalized screening. Models for prediction of breast cancer risks are now being developed to form a more comprehensive basis for personalized screening.^4^, ^5^ Besides breast density, these models include family history of breast cancer, life style factors, and genetic variants. Screening trials based on such models are currently under way as the American WISDOM study^6^ and the European MyPEBS study,^7^ where women are randomized to either a personalized, risk‐based screening strategy or to routine screening.

Here we investigated the potentials for personalized screening from a different perspective. We focused on the impact of health status on the actual outcome of screening, comparing women who had with women who had not been offered screening, stratified by health status. Several studies have shown that comorbidity increases mortality from breast cancer as well as all‐cause mortality.^8^, ^9^, ^10^ Women with severe comorbidities are less likely to receive appropriate treatment than women without comorbidities.^10^, ^11^ However, due to their comorbidities and consequently their high risk of dying from other causes of death, even appropriately treated women may not live long enough to benefit from early detection and treatment. In a recent study, Demb *et al* found that older, screened women with comorbidity had a higher risk of dying from other cause of death than older, screened women without comorbidity, but the risk of being diagnosed with or dying from breast cancer was the same in the two groups.^12^ These results suggested a limited benefit of breast cancer screening in older women with comorbidities, but the actual effect remains untested.

In the present study, we tested whether the presence of chronic disease modified the effect of screening on breast cancer mortality in women invited to population‐based screening in Denmark, using individual‐level data.

## 2 MATERIALS AND METHODS

### 2.1 Breast cancer screening program

In Denmark, organized breast cancer screening programs started in Copenhagen municipality on 1 April 1991 and in the county of Funen on 1 November 1993. These two pioneer programs covered 20% of Danish women and invited to screening every second year women aged 50 to 69 years. Screening in the rest of Denmark was implemented in 2007‐2008.

### 2.2 Study and control groups

**Figure 1:**
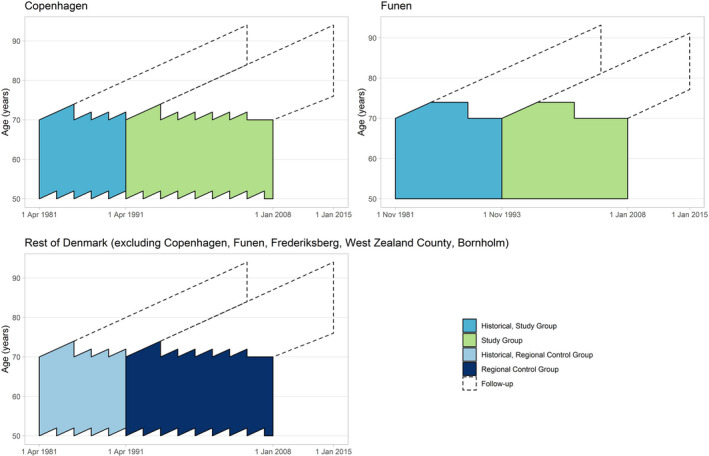
Study design illustrated in Lexis’ diagrams. The groups were constructed using the date of birth and current and historical addressees retrieved from the Danish Central Population Register. The areas surrounded by the solid line represent the (pseudo‐) screening period, and the areas surrounded by the dotted line the (pseudo‐) after screening period. The former and the latter represent the follow‐up period.

Thanks to the gradual implementation of breast cancer screening, Denmark can be seen as a “natural experiment” with screened and non‐screened regions (Table S1). Hence, this setting allowed for both regional and historical comparisons. Briefly, the design consisted of one study group of birth cohorts invited to screening; and of three control groups: a regional control group including the same birth cohorts from regions of Denmark where screening was not implemented; a historical control group including birth cohorts from the screening regions prior to screening; and a historical, regional control group including birth cohorts from the non‐screening regions in this historical period. Figure 1 represents the Lexis's diagrams for women included in the study.

The study group included women invited to screening in the Copenhagen pioneer program between 1 April 1991 and 31 December 2007, and women invited to the Funen pioneer program between 1 November 1993 and 31 December 2007. Women were included from their first date of invitation to screening. The regional control group included women living in non‐screening regions of Denmark during the same period. We followed women in these groups until 31 December 2014, except for women born after 31 December 1937 that were followed only until 31 December 2007, when they started to be invited in the national screening program (Figure 1, Method S1 and Table S2).

We used two historical control groups; first, women living prior to screening in Copenhagen between 1 April 1981 and 31 March 1991 or in Funen between 1 November 1981 and 31 October 1993 (the historical study group); second, women living in non‐screening regions (historical, regional control group) during the same period. We followed women in the historical groups until the start of the pioneer programs, that is 31 March 1991 for Copenhagen and until 31 October 1993 for Funen. However, women living in Copenhagen born before 1 April 1921 (and women living in Funen born before 1 November 1923) were followed until 31 December 2004, as these women were already above screening age when the national program started (Figure 1, Method S1 and Table S2).

We allocated a pseudo‐invitation date to women in the control groups, following the algorithm used for the study group. In all groups, we excluded women diagnosed with breast cancer before first invitation (pseudo‐invitation).

We used the three control groups to estimate the expected outcome in the absence of screening. The expected outcome in the absence of screening was estimated by the outcome in the non‐screening region (regional control group) controlling for historical differences between the outcome in the screening region (historical study group) and the outcome in the non‐screening region (historical, regional control group). In this way, we controlled for differences between regions and changes over time that could affect breast cancer mortality. This study design has been described in detail previously.^3^, ^13^

### 2.3 Data source

Individual data on vital status and current and historical addresses were retrieved from the Danish Central Population Register.^14^ We identified breast cancer diagnoses from the Danish Cancer Register,^15^ and underlying causes of death from the Danish Cause of Death Register.^16^ Data on dates of invitation and screening attendance were received from the screening registers in Copenhagen and Funen. We retrieved information on chronic diseases from the Danish National Patient Register,^17^ which includes diagnoses for all in‐patient contacts to Danish hospitals somatic wards since 1977, and for all out‐patient contacts, and in‐patient contacts to psychiatric wards since 1995. Diagnoses are classified according to the *International Classification of Disease*, 8th revision (ICD‐8) until the end of 1993 and 10th revision (ICD‐10) thereafter. We linked the data using the personal identification number assigned to all residents in Denmark.

### 2.4 Chronic diseases data

All discharge diagnoses (primary, secondary, and additional diagnoses) during the 4 years before invitation (pseudo‐invitation) to screening were used to detect the presence of chronic diseases included in the Charlson Comorbidity Index.^18^ These diseases included liver diseases, myocardial infarction, congestive heart failure, peripheral vascular disease, chronic pulmonary disease, cerebrovascular disease, hemiplegia, dementia, connective tissue disease, ulcer disease, diabetes, renal disease, cancer, and HIV/AIDS, see Table S2 in the Supplementary Data for ICD codes. We excluded codes for breast cancer because the population was breast cancer‐free at the start of follow‐up. Women were categorized as having no or one or more chronic diseases at beginning of follow‐up.

### 2.5 Outcomes

We assessed breast cancer mortality defined as death from breast cancer (ICD‐8; 174 and ICD‐10; C50, D50).

### 2.6 Statistical analysis

We tabulated women by age (50‐54, 55‐59, 60‐64, 64‐69, 70‐74) and prevalent chronic diseases at beginning of follow‐up.

To analyze the effect of invitation to screening, we modeled a Poisson regression comparing the observed breast cancer‐specific mortality rate in women invited to screening with the expected rate in the absence of screening,^3^ see Method S2 in the Supplementary Data. This analysis was made separately for women having no and for women having one or more chronic diseases.

We used the “Evaluation model” conceptualized by Nyström *et al* ^19^ and Beau *et al* ^3^ The model includes breast cancer deaths in women diagnosed with breast cancer during screening age (ie, we added 6 months to allow time for diagnosis), and equivalent for the control groups. For women of screening age, person‐years were accumulated from date of invitation (or pseudo‐invitation) until date of death, emigration, or end of follow‐up, whichever came first. For women after screening age, person‐years were accumulated in the same way but only for women with breast cancer diagnosed during screening age. This model minimizes the dilution of the screening effect from breast cancer deaths in patients diagnosed after end of screening age.^3^ We assessed the effect modification by health status using a likelihood ratio test.

We conducted supplementary analyses on women participating at first invitation to screening using the method by Duffy *et al*.^20^ The rate ratio for participants compared with that of nonparticipants was adjusted for the increased breast cancer mortality rate in nonparticipants calculated as: RR_2_ = $\frac{p\psi D_{r}}{1-\left(1-p\right)D_{r}}$ , where *p* denotes the proportion of participants at first invitation to screening, ψ is the estimated rate ratio for participants compared with nonparticipants, and *D_r_* is the rate ratio for nonparticipants compared with uninvited control groups.

Results were adjusted by current age (divided into 5‐year age group) and expressed as rate ratios (RR) with two‐sided and 95% confidence interval (CI). We analyzed data using SAS version 9.4 (SAS Institute Inc, Cary, NC).

It was not possible to calculate the power of the study beforehand as we did not know the size of the group of women having chronic diseases before we had linked and edited all the register data.

## 3 RESULTS

The study was based on 12 598 866 person‐years, 1 421 769 from women invited to screening, and 11 177 097 from control subjects.

Overall, the mean age at beginning of follow‐up was 57.0 ± 6.2 years. The prevalence of chronic diseases before first invitation (pseudo‐invitation) to screening was 7% to 9% (Table 1), with vascular disease, chronic pulmonary disease, and cancer (other than breast cancer) as the most common.

**Table 1:** Number of women invited to breast cancer screening in two early, organized programs in Denmark (study group), and number of women in three control groups; number of women by age, and number of women with chronic diseases

	Study Group (N = 181 299)	Regional Control Group (N = 822 370)	Historical Study Group (N = 162 518)	Historical, Regional Control Group (N = 541 558)
Age category at beginning of follow‐up, N(%)				
50‐54 yr	105 109 (57.98)^a^	517 886 (62.97)	63 700 (39.20)	242 211 (44.72)
55‐59 yr	25 464 (14.05)	101 036 (12.29)	29 406 (12.09)	101 037 (18.66)
60‐64 yr	23 793 (13.12)	93 279 (11.34)	32 470 (19.98)	95 106 (17.56)
65‐69 yr	23 464 (12.94)	90 063 (10.95)	32 066 (19.73)	84 357 (15.58)
70‐74 yr	3469 (1.91)	20 106 (2.44)	4876 (3.00)	18 847 (3.48)
Chronic diseases at beginning of follow‐up^b^, N (%)				
No chronic diseases	165 749 (91.42)	761 389 (92.58)	151 161 (93.01)	504 034 (93.07)
One or more chronic diseases	15 550 (8.58)	60 981 (7.42)	11 357 (6.99)	37 524 (6.93)
Vascular disease	4308 (2.38)	17 784 (2.16)	3318 (2.04)	11 523 (2.13)
Dementia	162 (0.09)	532 (0.06)	216 (0.13)	518 (0.10)
Chronic pulmonary disease	3358 (1.85)	12 199 (1.48)	1813 (1.12)	5991 (1.11)
Connective tissue disease	1784 (0.98)	7885 (0.96)	11 411 (0.87)	4628 (0.85)
Ulcer disease	1408 (0.78)	6086 (0.74)	1279 (0.79)	4488 (0.83)
Diabetes 1 and 2	2586 (1.43)	8852 (1.08)	1542 (0.95)	5469 (1.01)
Liver disease	844 (0.47)	2395 (0.29)	547 (0.34)	1267 (0.23)
Hemiplegia	61 (0.03)	336 (0.04)	83 (0.05)	286 (0.05)
Moderate to severe renal disease	411 (0.23)	1602 (0.19)	407 (0.25)	1554 (0.29)
Cancer^c^	3106 (1.71)	12 668 (1.54)	2600 (1.60)	8168 (1.51)
AIDS	35 (0.02)	34 (0.00)	0 (0.00)	^d^

^a^ 324 women were less than 50 years at invitation to screening.

^b^ During the 4 years before invitation (pseudo‐invitation) to screening, women can have more than one chronic disease.

^c^ Breast cancer diagnosis was not counted.

^d^ Three or less individuals.

For the study group as a whole, we observed a 26% reduction in breast cancer mortality after invitation to screening (age‐adjusted RR = 0.74 (95% CI, 0.66‐0.82); Table 2). However, the effect of invitation to screening on breast cancer mortality was only 7% in women with chronic diseases (age‐adjusted RR = 0.93 (95% CI, 0.63‐1.39)), whereas the effect was 28% in women without chronic diseases (age‐adjusted RR = 0.72 (95% CI, 0.65‐0.80)). The difference between the two groups of women did not reach statistical significance (*P*‐value for interaction = 0.22).

**Table 2:** Breast cancer deaths, person‐years, breast cancer mortality rates, and rate ratio estimates for the effect of screening on breast cancer mortality; all invited women, invited women without chronic diseases, and invited women with chronic diseases

	Study Group	Regional Control Group	Historical Study Group	Historical, Regional Control Group	Expected rate in the absence of screening^a^	Screening effect^b^	
	No. of breast cancer deaths/PY per 1,000/rate per 100 000 PY				Rate per 100 000 PY	Crude rate ratio (95% CI)	Age‐adjusted rate ratio^c^ (95% CI)
Overall	903/ 1421/ 63.5	4,848/ 6,733/ 72.0	888/ 1,082/ 82.0	2,255/3,361/67.1	88.0	0.72 (0.65‐0.80)	0.74 (0.66‐0.82)
No chronic diseases	821/ 1322/ 62.1	4,527/ 6,336/ 71.5	833/ 1,019/ 81.7	2,095/3,159/66.3	88.1	0.71 (0.63‐0.79)	0.72 (0.65‐0.80)
One or more chronic diseases	82/ 99/ 82.6	321/ 397/ 80.8	55/ 63/ 86.7	160/202/ 79.0	88.7	0.93 (0.63‐1.38)	0.93 (0.63‐1.39)

Abbreviations: CI, confidence interval; PY, person‐years.

^a^ Expected rate in the absence of screening = Regional Control Group x (Historical Study Group/Historical, Regional Control Group).

^b^ Screening effect = Study Group/Expected rate in the absence of screening.

^c^ Adjusted by current 5‐year age groups.

Participation at first invitation to screening was 71% among women with chronic diseases versus 77% among those without (Table 3). For women with chronic diseases before follow‐up, the RR of breast cancer mortality was 0.70 (95% CI, 0.43‐1.13) for participants at first invitation, the RR for nonparticipants was 1.25 (95% CI, 0.74‐2.09), and the corrected RR of dying from breast cancer for participants with chronic diseases became 0.96 (95% CI, 0.38‐2.46). For women without chronic diseases, the RR for participants was 0.51 (95% CI, 0.44‐0.59), and 1.21 (95% CI, 1.04‐1.40) for nonparticipants, and the RR of dying from breast cancer for participants without chronic diseases became 0.65 (95% CI, 0.51‐0.84). The difference between the two groups of participants did not reach statistical significance (*P*‐value for interaction = 0.43).

**Table 3:** Rate ratio estimates for the effect of screening on breast cancer mortality; all participating women after first invitation to screening, participating women without chronic diseases, and participating women with chronic diseases

	Participation in the first invitation to screening	Participants in the Study Group	Nonparticipants in the Study Group	Screening effect among participants^a^	Comparison between nonparticipants and noninvited control groups^b^	Screening effect among participants, corrected for selection bias^c^
	N (%)	No. of breast cancer deaths/PY per 1,000/ rate per 100 000 PY		Age‐adjusted rate ratio^d^ (95% CI)		
Overall	138,692 (76.50)	605/ 1123/ 53.8	298/ 298/ 100.0	0.52 (0.45‐0.60)	1.21 (1.05‐1.39)	0.67 (0.53‐0.86)
No chronic diseases	127,616 (76.99)	547/ 1048/ 52.2	274/ 275/ 99.7	0.51 (0.44‐0.59)	1.21 (1.04‐1.40)	0.65 (0.51‐0.84)
One or more chronic diseases	11,076 (71.23)	58/ 76/ 76.2	24/ 23/ 103.8	0.70 (0.43‐1.13)	1.25 (0.74‐2.09)	0.96 (0.38‐2.46)

Abbreviations: CI, confidence interval; PY, person‐years.

^a^ Described in Duffy et al^20^ as ψ , the RR of breast cancer mortality for participants in the first invitation to screening compared with nonparticipants.

^b^ Described in Duffy et al^20^ as *D_r_*, the RR of breast cancer mortality for nonparticipants compared with noninvited control groups.

^c^ Described in Duffy et al^20^ as RR_2_, the effect of offering screening to those who would participate if invited.

^d^ Adjusted by current 10‐year age group

## 4 DISCUSSION

We observed that invitation to organized, population‐based screening was associated with a reduction of only 7% in breast cancer mortality among women with chronic diseases, while a 28% reduction was observed among women without chronic diseases. Attendance to screening was lower in women with than in women without chronic diseases, but the difference in effect of screening among participants was even larger than for all invited women. After controlling for selection bias, participating women with chronic diseases experienced a 4% reduction in breast cancer mortality, while the reduction was 35% among participants without chronic diseases. The Danish screening programs invited women aged 50 to 69 years old, and only 9% of invited women suffered from chronic diseases. Despite the large difference observed in the effect of screening between women with and women without chronic diseases, our groups were too small for the difference to reach statistical significance.

A limited effectiveness of screening in women with chronic diseases might be expected. First, already before invitation to screening women with chronic diseases may be under clinical surveillance with the possibility of having early symptoms of breast cancer investigated. Nevertheless, we cannot separate out a possible effect of clinical surveillance from a possible effect of a higher breast cancer risk, for example in women with diabetes.^21^ Secondly, breast cancer patients with chronic diseases may have received less optimal treatment than women without chronic diseases, as clinicians may be concerned about the toxicity of treatment, treatment might be less effective, or life expectancy might be judged too short to justify treatments^10^, ^11^ Using Danish data, Land *et al* reported that breast cancer patients with comorbidities were less likely than those without to be enrolled in treatment protocols; 57% vs 76%.^22^ Thirdly, breast cancer patients with chronic diseases may have benefitted less from a given treatment than those without chronic diseases. The data on efficacy of breast cancer treatment derive mainly from randomized control trials where women with comorbidities are, mostly, excluded. However, using observational studies, Land et al showed that patients with comorbidities who actually received adjuvant treatment benefitted to the same extent as patients without comorbidity.^22^, ^23^ Finally, the effectiveness of screening depends on the sensitivity of the test. We estimated the sensitivity of the Copenhagen program as the proportion of screen‐detected cancer out of all breast cancer diagnosed within 24 months of the first invitation (or before the next screen). We found the sensitivity to be slightly lower, but not statistically different, among women with chronic diseases 56% (95% CI, 45%–66%) than for women without 66% (95% CI, 63%–70%). Sensitivity is often used as a short‐term indicator of screening effectiveness,^24^ and our results indicate that sensitivity by health status at invitation may be an indicator for the expected effect of screening on breast cancer mortality.

Age at screening has been a key topic in the investigation of differential effects of screening on breast cancer mortality,^1^ but few studies have focused on other factors. In a case‐control study nested in a population‐based screening program in Nijmegen, the Netherlands, Ripping et al^25^ found the reduction in breast cancer mortality to be slightly stronger, but nonsignificantly, in women with low than in women with high socioeconomic status, and Waal et al^26^ found a smaller breast cancer mortality reduction among women with dense than among women with fatty breasts. Demb et al estimated the 10‐year risk of invasive breast cancer, breast cancer death, and other cause of death according to comorbidity and age among women screened at ages 66 to 94 years without a history of breast cancer.^12^ As expected, the non‐breast cancer‐related mortality increased with increasing comorbidity. As expected, the breast cancer mortality was low in these women who were breast cancer‐free at the time of recruitment, and it did not vary across comorbidity group. These results support the findings from the Swedish randomized trials, showing that breast cancer deaths constitute only a small proportion of all deaths.^27^ The Demb et al data cannot directly be compared with our data because it include women screened up to age 94 years, which is far beyond the stop of screening at age 69 years in Denmark. Moreover, Demb et al did not test the differential effect of screening between women with and without chronic diseases.

For the population at large, a meta‐analysis of randomized control trials from the US and Sweden estimated that to prevent one breast cancer death per 1000 screened women, the women should have a remaining life expectancy of at least 10 years.^28^ Some US screening guidelines, for instance from the American Cancer Society,^29^ recommend stopping screening in women with a remaining life expectancy of less than 10 years, while Europe screening guidelines are based solely on age.^2^ In any case, assessing the remaining life expectancy of a woman is challenging. Hence, the burden of chronic diseases of an individual might be the best proxy for the remaining life expectancy. For instance, studies have used comorbidity scores to develop comorbidity‐adjusted life expectancy tables.^30^, ^31^ Moreover, mortality indices that take into account age and medical conditions have been used in clinical practice to predict the 4‐ to 10‐years mortality.^32^, ^33^ These studies highlight that the health status could help in tailoring cancer screening decisions. Our approach, based on the presence or absence of chronic diseases, might be a more direct way to distinguish women likely to benefit from screening from those not likely to benefit. Information on the presence of diseases is easily available either from asking the woman or, as in our case, from national health registers.

Without partitioning our population based on the presence or absence of chronic diseases prior to screening, we found a 26% breast cancer mortality reduction in women invited for screening and a 33% reduction in screened women. These results are in line with previous evidence as reported by the IARC report.^24^ Our analysis in women with chronic diseases at the time of invitation to screening showed little impact of screening in this population. This result is new, and further research is needed to test our findings. If similar patterns are observed, the presence of chronic diseases is an important factor to consider in personalized screening. Individualized decision‐making approaches, for instance, taking into account the severity of the chronic diseases, the likelihood to tolerate breast cancer treatment, the women's age and preferences, might be offered to women with chronic diseases to improve their potential benefit from screening and to optimize the health‐care resources.

Moreover, more knowledge of harms of screening is needed for building a complete picture of the screening balance for women with chronic diseases. In a modeling study using data from England and Norway, Falk and Hofvind estimated that in a population offered screening, between 2% and 4% of women diagnosed with breast cancer would die from other causes within the estimated lead‐time.^34^ Owing to the reduced life expectancy, we expect this inevitable proportion of overdiagnosed breast cancer cases to be larger in women with than in women without chronic diseases.

### 4.1 Limitations and strengths

Our study has limitations. The Charlson Comorbidity Index^18^ identified from hospital records may not capture all diseases associated with breast cancer mortality; some women with chronic diseases but without hospital contacts during the last 4 years may be misclassified to the no chronic diseases group. Thus, it underestimated the true difference between the two groups. In addition, our study was based on a young population at beginning of follow‐up limiting the size of the group of women with chronic diseases. A broad range of diseases is included in the Charlson Comorbidity Index; however, due to the relatively small size of the group of women with chronic diseases, we were not able to study subgroups. Lastly, the denominator in our calculation of the post‐screening rate of breast cancer mortality could potentially have been inflated if we had many nonlethal overdiagnosed breast cancer cases, but in Denmark, with sufficient time for post‐screening follow‐up, overdiagnosis was estimated to account for only 2.3% of breast cancer cases.^35^

The strengths of our study are the cohort design with inclusion of a study group invited to screening and three control groups not invited to screening, and use of individual linkage between registers with complete follow‐up of all groups. In our data, the study group includes only observations from women actually targeted by screening. This is in contrast to routine statistics data, like NORDCAN, where the tabulation by age groups will include observations from many women not targeted by screening. Opportunistic screening was rare in Denmark, ensuring that there was very little contamination of screening in the control groups.^36^ Data used to derive women's health status before follow‐up were from a national register with complete registration of hospital contacts. Furthermore, the Charlson Comorbidity Index is a validated tool, which has been used intensively, allowing for comparison of our findings with previous studies.

## 5 CONCLUSION

In conclusion, among women without chronic diseases, we observed a 28% reduction in breast cancer mortality following invitation to screening. However, the reduction was only 7% among women with chronic diseases. This difference was even larger among participants with a 35% reduction for women without and 4% for women with chronic diseases. Even though the differences between the groups did not reach statistical significance, our data indicate that not only the expected risk of breast cancer but also the expected benefit and harm from participating in screening might be considered in personalized screening.

## CONFLICT OF INTEREST

The authors have declared no conflict of interest.

## AUTHOR CONTRIBUTIONS

EL and ABB designed the study. AsBB, GMN, and EL collected and assembled the data. ABB performed the statistical analyses. GMN and PKA reviewed the statistical analyses. ABB, GMN, and EL had full access to all of the data in the analyses. ABB and EL drafted the manuscript. All authors interpreted the data and critically revised the manuscript for important intellectual content. All authors approved the final version of the manuscript to submit for publication.

## ETHICS

The study was approved by the Danish Data Protection Agency (No. 2015‐57‐0121).

## Supporting information

Supplementary Material

## Data Availability

Research data are not shared.
