# Supplementary material for: Impact of chronic diseases on effect of breast cancer screening
Source: Cancer Med. 2020 Apr 6;9(11):3995–4003. doi: 10.1002/cam4.3036 (PMC7286470; doi:10.1002/cam4.3036)
Supplement: Supplementary file 1 — Supplementary Material [file CAM4-9-3995-s001.docx]

**Supplementary**

**Method S1**

**Danish breast cancer screening program**

The Copenhagen screening program started on April 1, 1991. Women aged 50–69 years were invited every two years to screening. The Funen screening program started on 1 November 1993, offering biennial screening to women aged 50–69 years at the date of invitation. All targeted women were personally invited to participate.

Other regional, organized programs were implemented in the municipality of Frederiksberg in 1994, in Bornholm in 2001, and in part of the County of Vestsjælland in 2004. The national organized mammography screening in Denmark started in end 2007/2008.

**Group construction**

**Table S1.** **Danish natural experiment.**

| **Region** | **Time period** | |
| --- | --- | --- |
|  | **Before screening** | **During screening** |
| **Screening region**  **(Copenhagen and Funen)** | Historical study group | Study group |
| **Non-screening region**  **(Rest of Denmark)** | Historical, regional control group | Regional control group |

**Contemporary group - Study group**

We included women invited to screening in the Copenhagen program during April 1, 1991 – December 31, 2007 and women invited to the Funen program during November 1, 1993 – December 31, 2007. We combined into one study group women invited to one of the program. Once a woman was invited to screening, she remained in the study group even if she moved to another region.

**Contemporary group - Regional control group**

The regional control group included women living in (or moving to) a non-screening region (rest of Denmark excluding Copenhagen, Funen, Frederiksberg, Bornholm and Vestsjælland) during April 1, 1991 – December 31, 2007. A first pseudo-invitation date was allocated to each woman following the scheme similar to that of the Copenhagen program. We censored the women in the regional control group from their first invitation date in the study group and from date of moving to Frederiksberg, Vestsjælland and Bornholm at screening age and operational periods for these programs.

Women living in Frederiksberg, Bornholm and Vestsjælland at screening age and operational periods for these programs prior to their first (or pseudo-) invitation were excluded from the contemporary groups.

Women born after December 31, 1938 were invited to the national screening program from January 1, 2008, hence we followed them only until December 31, 2007. All other women were followed until December 31, 2014.

**Historical group – Historical, study group**

The historical, study group included women living in Copenhagen municipality during April 1, 1981 – March 31, 1991 and women living in Funen County during November 1, 1981 – October 31, 1993. Similarly to the study group, we combined into one historical, study group women living in Copenhagen and Funen. A first pseudo-invitation date was allocated to each woman following the scheme similar to that of the Copenhagen or Funen program.

**Historical group – Historical, regional control group**

The historical, regional control group included women living in (or moving to) non-screening region (rest of Denmark excluding Copenhagen, Funen, Frederiksberg, Vestsjælland and Bornholm) during April 1, 1981 – March 31, 1991. A first pseudo-invitation date was allocated to each woman following the scheme similar to that of the Copenhagen program. We censored the women from the historical, control group from their first pseudo- invitation date in the historical, study group.

Women born after April 1, 1921 and living in Copenhagen were invited to the Copenhagen screening program from April 1, 1991, hence we followed them only until March 31, 1991. Similarly, women born after November 1, 1923 and living in Funen County were invited to the Funen screening program from November 1, 1993, hence we followed them only until October 31, 1993. All other women were followed until December 31, 2004, ensuring comparable length of follow-up with the contemporary groups.

**Table S2. Birth cohorts and observation periods.**

|  |  | **Birth cohorts** | **Follow-up** |
| --- | --- | --- | --- |
| Copenhagen | Historical, study group | Apr 1, 1911 –  Mar 31, 1921 | Apr 1, 1981 –  Dec 31, 2004 |
|  |  | Apr 1, 1921 –  Mar 31, 1939 | Apr 1, 1977 –  Mar 31, 1991 |
|  | Study group | Apr 1, 1921 –  Dec 31, 1937 | Apr 1, 1991 –  Dec 31, 2014 |
|  |  | Jan 1, 1938 –  Dec 31, 1957 | Apr 1, 1991 –  Dec 31, 2007 |
| Funen | Historical, study group | Nov 1, 1911 –  Oct 31, 1923 | Nov 1, 1981 –  Dec 31, 2004 |
|  |  | Nov 1, 1923 –  Oct 31, 1943 | Nov 1, 1981 –  Oct 31, 1993 |
|  | Study group | Nov 1, 1923 –  Dec 31, 1937 | Nov 1, 1993 –  Dec 31, 2014 |
|  |  | Jan 1, 1938 –  Dec 31, 1957 | Nov 1, 1993 –  Dec 31, 2007 |
| Rest of Denmark *(excluding Copenhagen,*  *Funen, Frederiksberg, Vestsjælland and Bornholm)* | Historical, regional control group | Apr 1, 1911 –  Mar 31, 1921 | Apr 1, 1981 –  Dec 31, 2004 |
|  |  | Apr 1, 1921 –  Mar 31, 1939 | Apr 1, 1981 –  Mar 31, 1991 |
|  | Regional control group | Apr 1, 1921 –  Dec 31, 1937 | Apr 1, 1991 –  Dec 31, 2014 |
|  |  | Jan 1, 1938 –  Dec 31, 1957 | Apr 1, 1991 –  Dec 31, 2007 |

**Table S2. ICD codes for identification of the Charlson conditions excluding breast cancer codes.**

| **19 Charlson Conditions** | **11 Modified Charlson Conditions** | **ICD-8** | **ICD-10** |
| --- | --- | --- | --- |
| Myocardial infarction | Vascular disease | 410; 427.09–427.11; 427.19; 428.99; 782.49; 440–445; 430–438 | I21–I23; I50; I11.0; I13.0; I13.2; I70–I74; I77; I60–I69; G45; G46 |
| Congestive heart failure |  |  |  |
| Peripheral vascular disease |  |  |  |
| Cerebrovascular disease |  |  |  |
| Dementia | Dementia | 290.09–290.19; 293.09 | F00–F03; F05.1;G30 |
| Chronic pulmonary disease | Chronic pulmonary disease | 490–493; 515–518 | J40–J47; J60–J67; J68.4; J70.1; J70.3; J84.1; J92.0; J96.1; J98.2; J98.3 |
| Connective tissue disease | Connective tissue disease | 712; 716; 734; 446; 135.99 | M05; M06; M08; M09; M30–M36; D86 |
| Ulcer disease | Ulcer disease | 530.91; 530.98; 531–534 | K22.1; K25–K28 |
| Mild liver disease | Liver disease | 571;573.01; 573.04; 070.00; 070.02; 070.04; 070.06; 070.08; 573.00; 456.00–456.09 | B18; K70.0–K70.3; K70.9; K71; K73; K74; K76.0; B15.0; B16.0; B16.2; B19.0; K70.4; K72; K76.6; I85 |
| Moderate to severe liver disease |  |  |  |
| Diabetes 1 and 2 | Diabetes 1 and 2 | 249.00–249.09; 250.00–250.09 | E10.0–E10.9; E11.0–E11.9 |
| Diabetes with end organ damage |  |  |  |
| Hemiplegia | Hemiplegia | 344 | G81; G82 |
| Moderate to severe renal disease | Moderate to severe renal disease | 403; 404; 580–584; 590.09; 593.19; 753.10–753.19; 792 | I12; I13; N00–N05; N07; N11; N14; N17-N19; Q61 |
| Tumor | Cancer (excluding breast cancer) | 140-194 (excluding 174) ; 204-207; 20-203; 275.59; 195-198; 199 | C00-C75 (excluding C50) ; C91-C95; C81-C85; C88; C90; C96; C76–C80 |
| Leukemia |  |  |  |
| Lymphoma |  |  |  |
| Metastatic solid tumor |  |  |  |
| AIDS | AIDS | 0.79.83 | B21–B24 |

**Method S2**

The expected breast cancer mortality in the absence of screening was estimated by the breast cancer mortality in a non-screening region (regional control group) controlling for historical differences in breast cancer mortality in a screening region (historical study group) and a non-screening region (historical, regional control group). Thus, the effect of screening was estimated by the ratio between the observed breast cancer mortality rate in the study group and the one expected in the absence of screening. This is a way to indirectly control for other factors varying between screening and non-screening regions over calendar time. The analysis would produce a valid estimate of the effect of screening under the assumption that in the absence of screening, the breast cancer mortality changed identically in screening and non-screening regions between the two time periods, i.e. no interaction between region and period. This is the “difference-in-differences” methodology widely used in econometrics.

This analysis was made separately for women having no and for women having one or more chronic diseases.
